# Multi‐Species Canopy Latrines in Costa Rican Cloud Forests: A Mammal Interactions Hub in a Single Tree Species

**DOI:** 10.1002/ece3.72964

**Published:** 2026-03-16

**Authors:** Jeremy Quirós‐Navarro, Tim Chamberlain, Deiver Espinoza

**Affiliations:** ^1^ Department of Ecology and Evolutionary Biology University of Connecticut Mansfield Connecticut USA; ^2^ Monteverde Biological Station Puntarenas Costa Rica; ^3^ Independent Researcher UK; ^4^ Independent Researcher Costa Rica

**Keywords:** animal behavior, arboreal mammals, canopy, *Ficus*, latrine, plant–animal interactions, tree climbing

## Abstract

We report the discovery of arboreal multi‐species mammal latrines in montane cloud forests of Costa Rica. We surveyed 169 trees from 29 species. Canopy multi‐species latrines were only found in 11 individuals of a single tree species, *Ficus tuerckheimii.* Camera traps recorded 17 mammal species and a total of 181 visits over 60 days, indicating that some vertebrates frequently visit canopy latrines. Among the most notable visitors was the two‐toed sloth ( *Choloepus hoffmanni* ), a species long documented to descend to the ground exclusively to defecate. Our findings suggest that sloths may also use arboreal latrines, challenging a long‐standing assumption in sloth ecology and raising new questions about the drivers of their defecation behavior. As with terrestrial latrines, canopy latrines may also play a role in interspecific communication, provide spatial cues, and affect nutrient dynamics in forest canopies. All these aspects highlight the potentially important role that *Ficus tuerckheimii* might have in these interaction points.

## Graphical abstract

We documented arboreal, multi‐species mammal latrines in montane cloud forests of Costa Rica by surveying 169 trees across 29 species. Latrines were found exclusively on Ficus tuerckheimii, with 11 occurrences across two mountain ranges and an additional observation in Honduras. Camera traps placed on one latrine recorded 17 mammal species visiting, providing insight into canopy defecation behavior and species interactions.

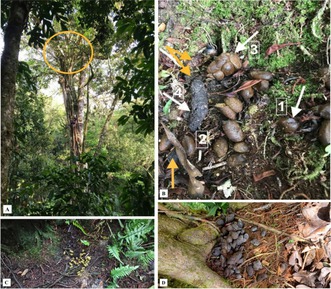

## RESUMEN

Reportamos el descubrimiento de letrinas arbóreas de mamíferos de múltiples especies en los bosques nubosos montanos de Costa Rica. Se censaron 169 árboles de 29 especies. Las letrinas multi‐especie de dosel solo se encontraron en 11 individuos de una única especie de árbol, *Ficus tuerckheimii.* Las cámaras trampa registraron 17 especies de mamíferos y un total de 181 visitas en 60 días, lo que muestra que algunos vertebrados visitan frecuentemente las letrinas del dosel. Entre los visitantes más notables está el oso perezoso de dos dedos ( *Choloepus hoffmanni* ), una especie que durante mucho tiempo se ha documentado que desciende al suelo exclusivamente para defecar. Nuestros hallazgos sugieren que los perezosos también podrían utilizar letrinas arbóreas, lo que desafía una suposición de larga data en la ecología de la especie y plantea nuevas preguntas sobre los factores que impulsan su comportamiento de defecación. Al igual que las letrinas terrestres, las letrinas del dosel también pueden desempeñar un papel en la comunicación interespecífica, en las señales espaciales y, además, en la dinámica de nutrientes en los doseles forestales. Todos estos aspectos resaltan la importancia del rol que puede tener *Ficus tuerckheimii* en estos puntos de interacción.

## 1 Introduction

Studying plant–animal interactions in tropical forest canopies is challenging. The first obstacle is climbing trees, since some reach heights up to 40–60 m tall. They are commonly covered in dense layers of epiphytes associated with harmful animals (e.g., wasps, vipers, ants) and bear many snap‐prone branches. As a result, tropical forest canopies remain a relatively understudied component of these ecosystems (Nakamura et al. 2022). The development and popularization of tree‐climbing techniques now makes possible ecological research in these upper forest strata, revealing many undocumented interactions and processes, such as nutrient dynamics (Lowman 2009; Nadkarni et al. 2011) and more recently, arboreal mammals ecology (Cudney‐Valenzuela et al. 2022; Moore et al. 2021).

One of those previously undocumented ecological features from the canopy is the mammal latrines. Animal latrines are defecation sites used repeatedly by one or multiple animals (Aadrean and Usio 2017; Brown 1979). Potential functions of latrines include territory defense, intra‐ and inter‐specific information exchange (e.g., reproductive signals and individual identity), facilitation of spatial orientation, and restriction of the spread of parasites (Nicolaides et al. 2024; Rodgers et al. 2015; Ziege et al. 2016). In addition to these communicative and ecological functions, latrines can also act as sites of encounter for predators and prey (Buesching and Jordan 2019). In a broader ecological sense, latrines can also serve as important nutrient sources, providing nitrogen to plants and influencing nutrients and plant spatial distribution through seed dispersal (Feeley 2005).

The tropical montane cloud forests in Central America are biodiversity hotspots and centers of endemism for many taxonomic groups (Anderson and Ashe 2000; Bruijnzeel et al. 2010; Bubb et al. 2004; Leo 1995; Long 1995; Stadtmüller 1987). One of the main trees of cloud forests is the fig tree (*Ficus* spp., Moraceae) (Burger 1977), described as a keystone species in tropical ecosystems (Bleher et al. 2003). These trees play fundamental roles as a food source, as hubs for birds that facilitate seed dispersal, and as shelter for animals in the numerous cavities found in mature trunks (Cottee‐Jones et al. 2016; Tello 2003).

Although in some terrestrial animals, such as gazelles or genets, the selection of latrine sites has been shown to be influenced by factors such as predator presence, habitat selection, understory height, and topography (Espírito‐Santo et al. 2007; Louhichi et al. 2025), it remains unclear how canopy‐dwelling mammals choose the location of their latrines. Our findings suggest a previously undocumented ecological role of *Ficus tuerckheimii*, as multi‐species latrines were consistently located in this tree species, considered a dominant cloud forest tree (Haber and W Bello 2000). This highlights the potential importance of tree architecture and spatial distribution in shaping latrine placement in the canopy.

All previous latrine reports in the literature are for terrestrial sites, often located near tree buttresses (Gonzalez‐Zamora et al. 2012) or close to human‐made structures (King et al. 2017). It has been assumed that canopy mammals do not use latrines, and feces are just dropped from tree branches (Voirin et al. 2013). Other mammals, such as sloths, are known to descend and defecate at the base of trees (Voirin et al. 2013). The objective of this study is to document, to date, the occurrence of multi‐species canopy latrines in cloud‐forest trees and, through the use of canopy camera traps, to identify the mammal species that visit and share these latrines.

## 2 Methods

### 2.1 Study Sites

All sampled trees are distributed across three mountain ranges in Costa Rica: the Central Volcanic Mountain Range, the Tilarán Mountain Range, and the Talamanca Mountain Range. The study sites span elevations from 1500 to 2000 m a.s.l., reaching up to 3200 m a.s.l. in the Talamanca Range. These regions are classified as montane cloud forests, receiving 2500–5000 mm of annual precipitation, with temperatures ranging from 12°C to 20°C and dropping to around 2°C during certain seasons, particularly at higher elevations. Camera traps for this study were deployed in a single latrine located in the Monteverde region of the Tilarán Mountain Range.

### 2.2 Survey of Canopy Latrines

From 2017 to 2024, as part of ongoing canopy research, we accessed trees using standard Single Rope Technique (SRT) methods for canopy exploration (Houle et al. 2004), following the protocols described by Lowman (2009) and Moore et al. (2021). To inspect the main branch–stem junctions and secondary bifurcations, we used lanyards to move safely along lateral and upper branches. Dead or structurally compromised trees were excluded to minimize risk during canopy access. We used basic certified tree climbing gear, including harnesses, ropes, carabiners, and helmets.

Tree selection focused on identifying individuals with horizontal branches connected to neighboring trees, maximizing the likelihood of detecting mammal activity. Over the years, we have observed that such interconnected trees function as canopy pathways, increasing the probability of animals using them (Quiros‐Navarro, J., unpublished data, 2020). This approach follows the same principle used in ground‐level camera trapping, where cameras are placed along animal trails to enhance detection probability (Fonteyn et al. 2021). Latrines were defined as defecation sites used repeatedly by one or multiple animals (Brown 1979).

### 2.3 Recording Mammal Detections at the Canopy Latrine

To determine the mammal species visiting canopy latrines, we installed two motion‐triggered camera traps recording video footage at a single latrine (NEXCAM Solar Trail Camera Model TC02). Batteries and memory cards did not need to be replaced during the study period. The latrine was selected as a known, active latrine site and was safely accessible for multiple visits. For this study, we monitored only one latrine; no additional latrines were equipped with cameras. The camera traps were powered by solar panels to minimize the need for frequent climbs and thereby reduce potential disturbance to the recordings. Both cameras ran simultaneously for 1440 camera‐hours (January—February 2024), and we recorded the species of mammals visiting the latrine. Mammals were identified using the camera videos and Costa Rican mammal field guides (Wainwright and Arias 2007). Where night‐time footage made identification more challenging, we compared it to day‐time imagery and examined morphological features and canopy‐use behavior to determine the species.

**FIGURE 1:**
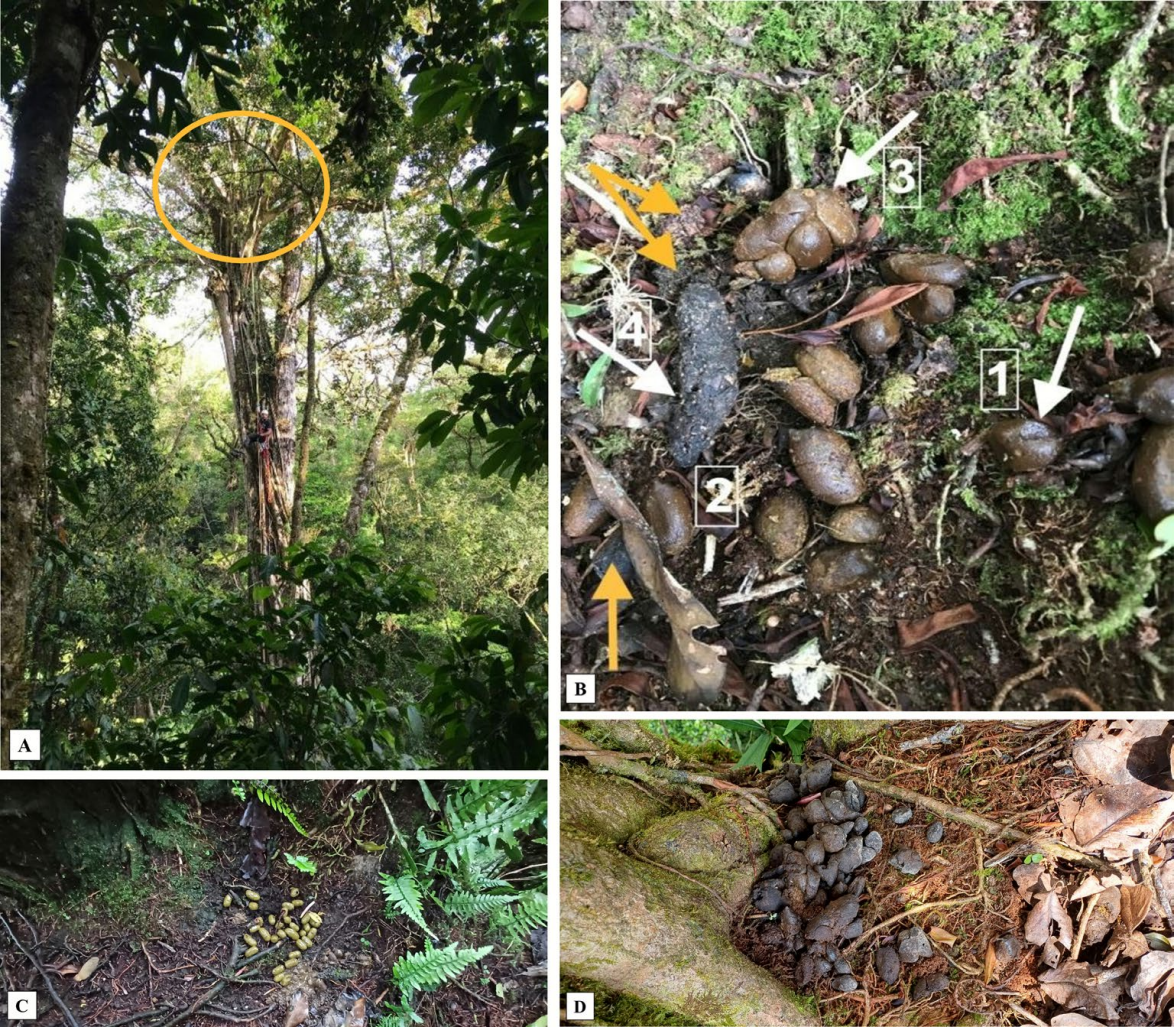
Canopy latrine in *Ficus tuerckheimii*. (A) Location of the canopy latrine used for this study, 30 m above ground. The circle indicates the latrine's position at the main branch union. (B) Feces from multiple species at the latrine. Numbers 1, 2 and 3 show two‐toed sloth feces ( *Choloepus hoffmanni* ). Number 4 shows what appears to be a carnivorous mammal. Yellow arrows indicate additional feces without identification. (C) Fresh feces from a porcupine (*Coendou mexicanus*) (deposition event observed on camera). (D) Old feces from a two‐toed sloth ( *Choloepus hoffmanni* ).

We also photographed the feces to help infer the diversity of animals using the latrines. The most abundant feces in the latrines appeared to show the distinctive morphologies associated with the orders Rodentia, Xenarthra, and families such as Mustelidae. We estimated that the most common species using canopy latrines include the Cacomixtle ( *Bassariscus sumichrasti* ), Hoffmann's two‐toed sloth ( *Choloepus hoffmanni* ), Kinkajou ( *Potos flavus* ), Mexican hairy dwarf porcupine (*Coendou mexicanus*), and Tayra ( *Eira barbara* ). However, in this study, species identification was based solely on camera‐trap evidence. This is because, although the morphology of some feces can be very distinctive (e.g., two‐toed sloths, Figure 1B,D), the identification of many mammal species would be challenging and tentative (Roland Kays, personal communication, December 6, 2023). The first camera was positioned above the latrine, and the second was oriented to the main connecting branch of the latrine. The second camera was installed to provide a different perspective of the mammals to assist in the identification, while the first camera monitors whether the animals interact or use the latrine. Detections were considered independent events only when separated by at least 30 min (O'Brien et al. 2003). We calculated the species occurrence of events per 100 days of camera trapping (Comley et al. 2023), and how many times each mammal uses the latrine by defecating or urinating on it.

## 3 Results

We climbed a total of 169 trees from 29 species. *F. tuerckheimii* is the only tree species in which we observed multi‐species latrines. Of the 15 *F. tuerckheimii* individuals surveyed, 11 had latrines (Table 1). All the latrines were located at the main bifurcation of the 11 latrine trees, which form a flat, open platform covered with moss, a thick layer of soil, fallen leaves, and other organic detritus (Figure 1). The monitored latrine was located at a height of 30 m above ground.

**TABLE 1:** Tree species in the survey of canopy latrines.

Species	Sample size	Ocurrence (%)
*Alnus acuminata*	10	0
*Beilschmiedia costaricensis*	3	0
*Billia rosea*	10	0
*Brunellia costaricensis*	7	0
*Cedrela tonduzii*	7	0
*Cinnamomun hammelianum*	3	0
*Citharexylum donell‐smithii*	5	0
*Citharexylum macradenium*	6	0
*Cleyera theaeoides*	1	0
*Croton draco*	4	0
*Damburneya cufodontisii*	3	0
*Ficus pertusa*	3	0
*Ficus tuerckheimii*	**15**	**73.33**
*Guarea kunthiana*	3	0
*Magnolia poasana*	7	0
*Panopsis suaveolens*	5	0
*Persea caerulea*	10	0
*Persea schiedeana*	2	0
*Prunus rhamnoides*	4	0
*Quercus bumelioides*	10	0
*Quercus cortesii*	2	0
*Quercus costaricensis*	7	0
*Quercus lancifolia*	13	0
*Quercus rapurahuensis*	5	0
*Quercus salicifolia*	10	0
*Sapium glandulosum*	3	0
*Sapium pachystachys*	3	0
*Sideroxylon portoricense*	4	0
*Ulmus mexicana*	4	0

*Note:* Note that latrines were observed in 11 of 15 sampled strangler fig trees (*Ficus tuerckheimii*), accounting for 73% of all sampled Ficus individuals. In contrast, none of the 154 individuals from the other 28 tree species showed any signs of latrine formation (0%). Bold values indicate that Fisher's exact test: *P* < 0.001.

**FIGURE 2:**
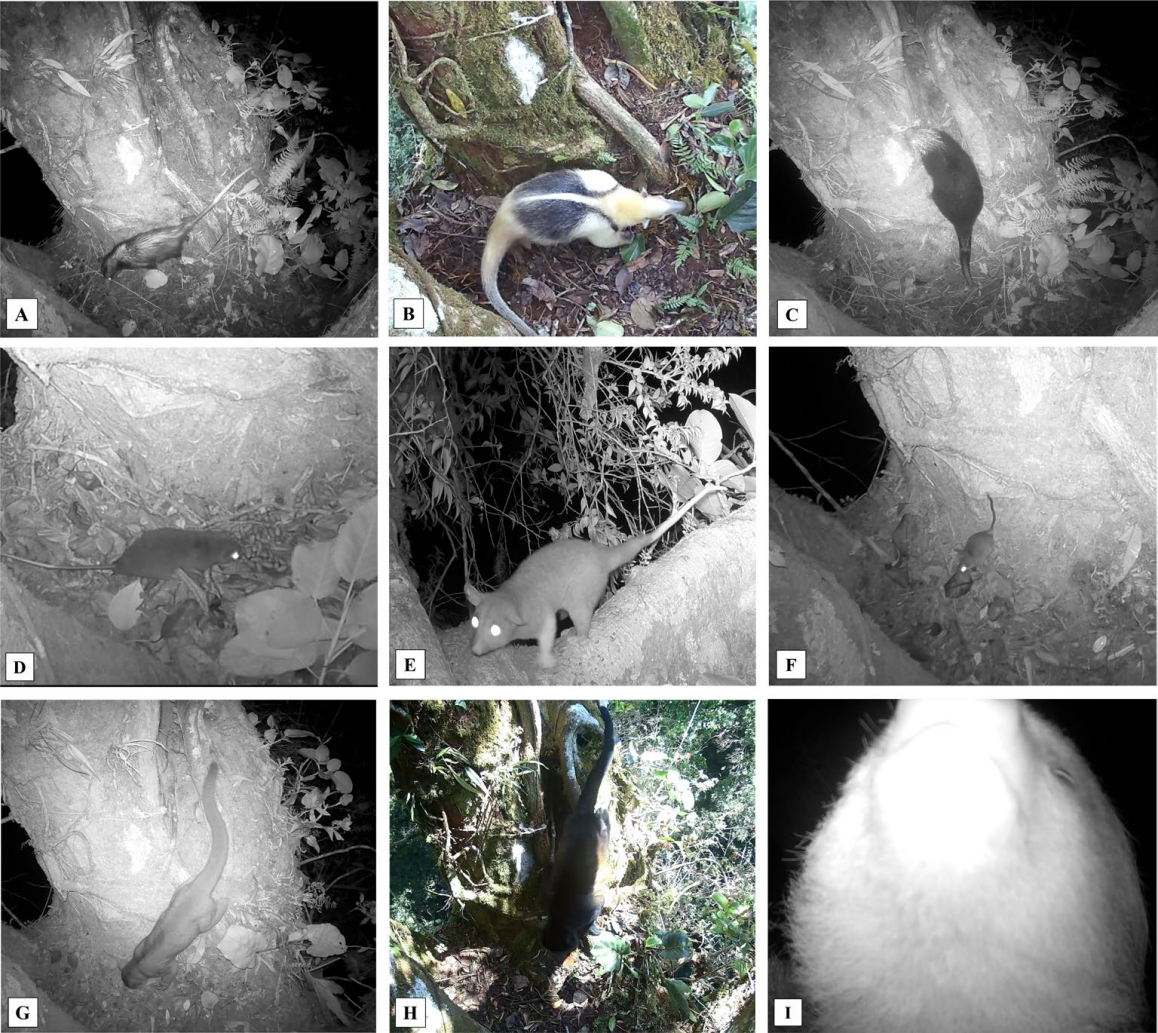
Still frames from video footage: Mammals visiting the latrine. (A) *Didelphis marsupialis*. (B) *Tamandua mexicana* . (C) *Coendou mexicanus*. (D) *Tylomys watsoni* . (E) *Caluromys derbianus* . (F) *Nyctomys sumichrasti* . (G) *Potos flavus* . (H) *Alouatta palliata* . (I) *Choloepus hoffmanni* .

**FIGURE 3:**
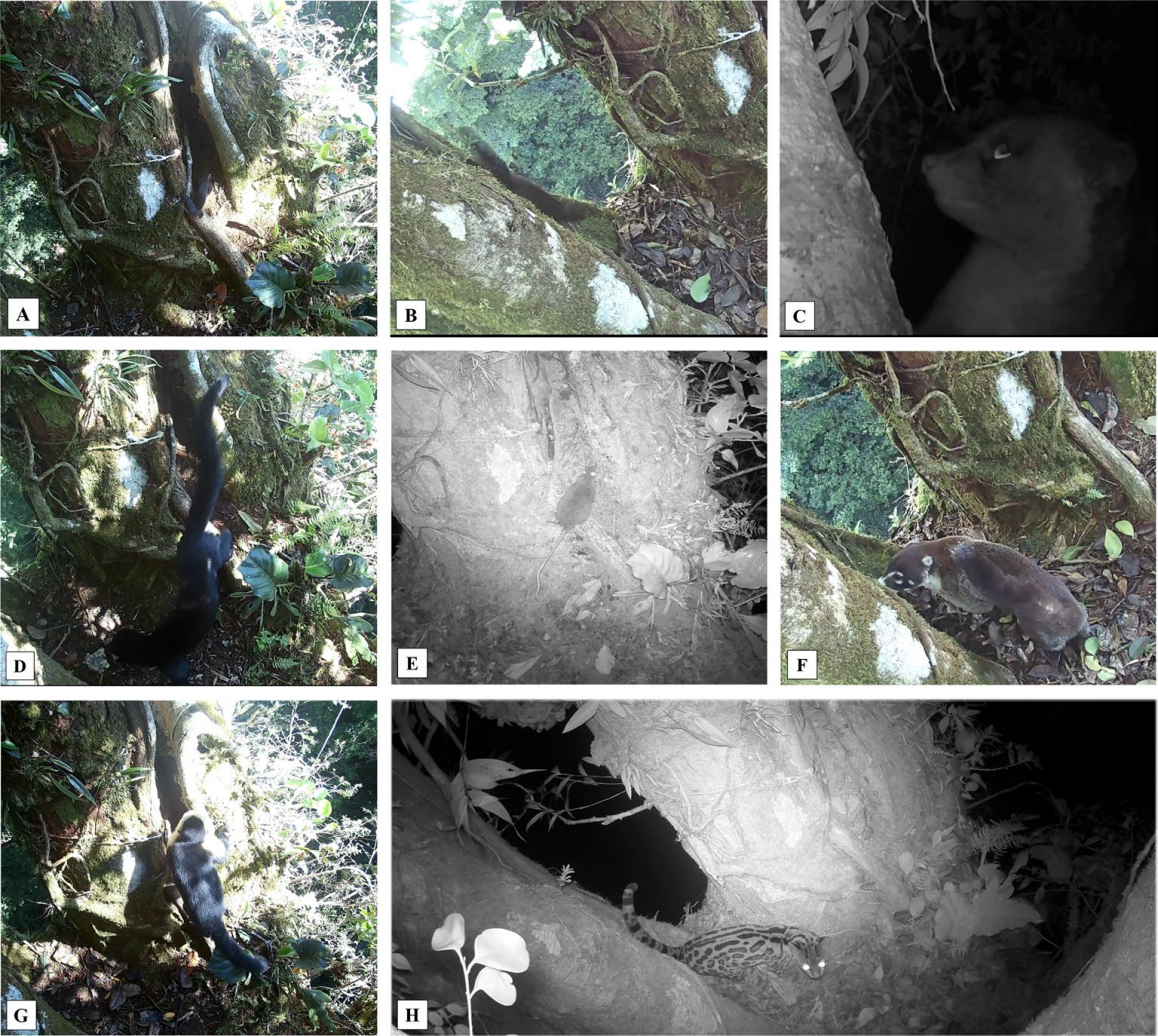
Still frames from video footage: Mammals visiting the latrine. (A) *Mustela frenata* . (B) *Sciurus granatensis* . (C) *Bassaricyon gabbii* . (D) *Eira barbara* . (E) *Liomys salvini* . (F) *Nasua narica* . (G) *Cebus capucinus* . (H) *Leopardus wiedii* .

**FIGURE 4:**
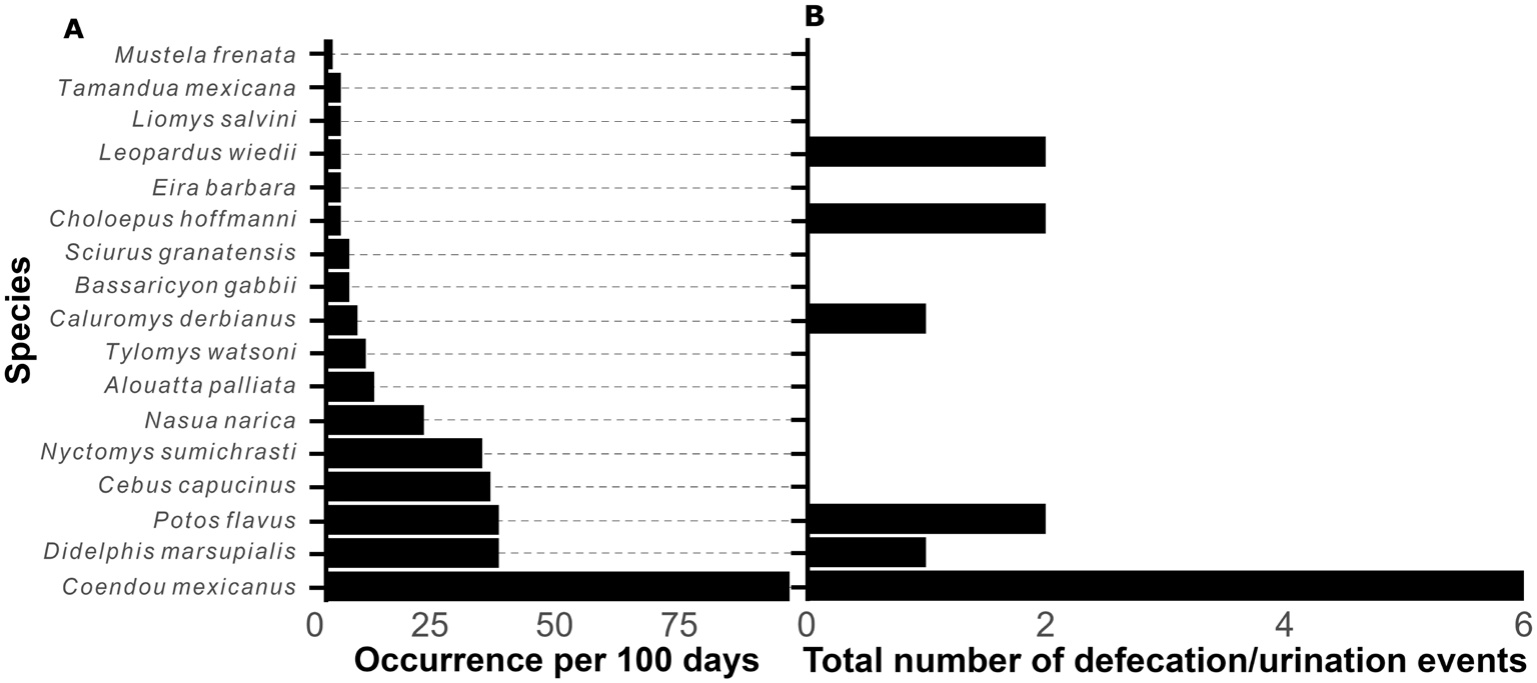
Graph (A) shows the frequency of occurrence of different mammal species at the site. Graph (B) shows the number of events when these mammals were recorded defecating/urinating at the latrine. Note that the margay observed ( *Leopardus wiedii* ) only urinated and did not defecate.

A total of 181 detections and 17 mammal species were recorded using or interacting with the latrine (Figures 2 and 3). The cameras recorded an average of 3.02 visits per day with no interspecific encounters observed. There were no days without at least one detection recorded. Regarding species occurrence, the animal that visited the latrine most frequently was the porcupine ( *C. mexicanus* ), followed by the opossum ( *Didelphis marsupialis* ) and kinkajou ( *P. flavus* ) (see all species occurrence in Figure 4A). When considering actual defecation or urination events, the porcupine ( *C. mexicanus* ) was again the most frequent user (Figure 4B). These patterns indicate both species diversity and differential frequency of latrine use among canopy mammals (Figure 4B).

Kinkajous and porcupines not only defecated or urinated on the latrine but also displayed rubbing behavior. Margays did not defecate but instead sprayed urine to mark the latrine. The videos also showed that they normally rested close to the latrine for several minutes. The two sloths recorded in the videos were females, as the individuals were observed carrying infants.

## 4 Discussion

Repeated observations across tree canopies revealed locations exhibiting what can be characterized as “clumped dung depositions”(dos Santos Neves et al. 2010), and repeated use of the same defecation sites (Buesching and Jordan 2019), two structural features consistent with animal latrines. The use of these sites by multiple species was demonstrated by camera‐trap records. Camera data showed repeated visits by different mammal species for defecation and urination at the same locations. Together, these observations support the classification of these sites as multi‐species animal latrines. To our knowledge, previous studies using camera traps to study latrines or fecal deposition sites have focused on ground‐level sites. The use of camera trapping to document latrine use in the forest canopy has not been previously reported.

Over 8 years of fieldwork, we climbed 169 canopy trees from the cloud forests of Costa Rica (Table 1). Although the sampling was not systematic, 11 latrines were found in only a single tree species. Trees listed in Table 1 represent a wide diversity of species and architectures, including large, medium, and small trees, as well as some fruit‐producing species that are frequently used by mammals. Despite this broad range of tree types surveyed, latrines were found exclusively in *F. tuerckheimii*. The presence of multi‐species canopy latrines only in trees of *F. tuerckheimii* across different cloud forests in Costa Rica, together with our recent discovery of canopy latrines in the same tree species in Honduras (Chamberlain, T. O., unpublished data, 2023), suggests that this is not a local phenomenon.

Our preliminary observations, based on camera‐trap monitoring at a single canopy latrine, have yielded valuable and important new insights that have motivated the implementation of the more extensive monitoring currently underway. Beyond documenting species richness, our findings provide novel insights into the use of latrines by canopy‐dwelling mammals. First, our evidence demonstrates that mammals inhabiting the forest canopy also establish and use latrines, and that these sites are shared by multiple species. Second, despite surveying a wide diversity of tree species, latrines were consistently associated with *F. tuerckheimii*, suggesting a strong and non‐random selection of this tree species for latrine establishment, although the ecological drivers underlying this preference remain unknown.

Although we identified 17 mammal species interacting with the latrine site where we deployed the cameras, notably, across the 181 recorded videos, there was no overlap in visitation. Each species appeared to visit the site independently, with no recorded interspecific encounters, which may indicate temporal partitioning or avoidance among species. While previous studies suggest that mammal visitation may be influenced by prey or predator presence (Harris et al. 2015), our limited dataset does not allow us to explore the drivers of this behavior, especially given that most latrine visitors are not predators, except for the olingo ( *Bassaricyon gabbii* ) and the margay ( *L. wiedii* ).

Importantly, camera‐trap evidence shows that species such as Hoffmann's two‐toed sloth ( *C. hoffmanni* ), previously assumed to always descend to the ground to defecate (Voirin et al. 2013), may also use latrines located in the canopy. Here, only female sloths were recorded using the latrine. Females were observed carrying infants, which suggests that defecation from the treetops may function as an anti‐predator behavior during maternal care. In addition, these latrine sites appear to function as communication or convergence points for canopy mammals. Camera data show that porcupines and kinkajous not only defecated or urinated on the latrine but also displayed rubbing behavior. The scent‐rubbing behavior has been broadly reported as a mechanism used by mammals for intraspecific communication through chemical signals (Gosling and McKay 1990).

Of particular relevance is the frequent visitation by the margay ( *L. wiedii* ). This is a highly arboreal felid that is difficult to monitor on the ground due to low detection rates, likely caused by its arboreal behavior (Harmsen et al. 2021). Detection in the canopy has also proven difficult (Quiros‐Navarro, J. & Espinoza, D., unpublished data, 2025). They were repeatedly observed visiting the site, not to defecate, but to spray urine, suggesting a potential territorial or communicative function of the latrine for this species (Chame 2003). Our results suggest that canopy latrines in *F. tuerckheimii* may represent strategic sites for future studies using camera traps aimed at improving our understanding of margay ecology, movement patterns, and habitat use in the forest canopy.

Latrines are known to serve multiple ecological functions. They are crucial for communication among many mammalian species (King et al. 2017; Stewart et al. 2002), acting as key sites for social interaction, even in largely solitary species like sloths (Kaup et al. 2021). Arboreal latrines may therefore play a crucial role in both intra‐ and inter‐specific communication.

In addition, they may provide supplemental nutrients to visiting species (Voirin et al. 2013) and may help reduce parasite loads (Gilbert 1997) however, increased parasite transmission among visitors remains an open question and represents an interesting direction for future research.

Furthermore, they could serve as focal gathering points for a wide range of taxa. For example, 14 mammal species have been documented visiting ocelot ( *Leopardus pardalis* ) ground latrines (King et al. 2017). These diverse functions highlight the potential ecological complexity of latrines, including those located in the canopy, and underscore the need for further research. Moreover, it is worth studying whether non‐focal species (e.g., insects, reptiles, and amphibians) also utilize these arboreal sites.

The reason these fig trees host more latrines than other species remains unclear. Nevertheless, we observed some distinct structural features; they are notably taller, have greater canopy connectivity and diameter, form a platform in the main bifurcation, and differ in overall architecture compared to surrounding trees. Additionally, we observed that many of these trees are often found along riverbanks, possibly serving as natural bridges connecting the two sides of the river. Taken together, our observations suggest that *F. tuerckheimii* may serve as a keystone structure in the canopy, supporting interaction and communication among arboreal mammals. Further empirical research is underway to compare characteristics of latrine trees versus non‐latrine trees and the relationship of these characteristics with mammal latrine preference.

Beyond their ecological significance, these findings may carry important implications for conservation management. Areas impacted by timber extraction (Felton et al. 2013) or experiencing growth in adventure tourism, including recreational tree climbing (McDermott 2022), could potentially disrupt the integrity of these canopy communication hubs. In the cloud forests of Costa Rica, *F. tuerckheimii* is commonly used by tourism companies to practice climbing their trunks. Given the ecological and functional importance of these trees as interaction sites for canopy‐dwelling species, such activities may inadvertently affect the integrity and continued use of these sites.

## Author Contributions

**Jeremy Quirós‐Navarro:** conceptualization (equal), data curation (lead), formal analysis (lead), funding acquisition (equal), investigation (lead), methodology (equal), project administration (equal), resources (equal), software (equal), supervision (equal), validation (equal), visualization (lead), writing – original draft (lead), writing – review and editing (lead). **Tim Chamberlain:** conceptualization (equal), data curation (supporting), formal analysis (supporting), funding acquisition (equal), investigation (equal), methodology (equal), project administration (equal), resources (equal), software (equal), supervision (equal), validation (equal), visualization (supporting), writing – original draft (supporting), writing – review and editing (supporting). **Deiver Espinoza:** conceptualization (equal), data curation (supporting), formal analysis (supporting), funding acquisition (equal), investigation (equal), methodology (equal), project administration (supporting), software (supporting), supervision (supporting), validation (supporting), visualization (supporting), writing – original draft (supporting), writing – review and editing (supporting).

## Conflicts of Interest

This manuscript is not under consideration by any other journal. All authors have read and approved the manuscript and declare no conflicts of interest.

## Data Availability

All the data that supports this study is available in the following link: https://doi.org/10.5061/dryad.j0zpc86tz.
